# Impact of melatonin administration on sperm quality, steroid hormone levels, and testicular blood flow parameters in small ruminants: A meta-analysis

**DOI:** 10.14202/vetworld.2024.911-921

**Published:** 2024-04-25

**Authors:** Agung Budiyanto, Slamet Hartanto, Rini Widayanti, Heri Kurnianto, Wardi Wardi, Bambang Haryanto, Ivan Mambaul Munir, Alek Ibrahim, Dini Dwi Ludfiani

**Affiliations:** 1 Department of Reproduction, Obstetrics, and Gynecology, Faculty of Veterinary Medicine, Gadjah Mada University, Yogyakarta, Indonesia; 2 Research Center for Animal Husbandry, National Research and Innovation Agency, Bogor, Indonesia; 3 Department of Biochemistry and Molecular Biology, Faculty of Veterinary Medicine, Gadjah Mada University, Yogyakarta, Indonesia; 4 Research Center for Veterinary Science, National Research and Innovation Agency, Bogor, Indonesia; 5 Research Center for Sustainable Production System and Life Cycle Assessment, National Research and Innovation Agency, Banten, Indonesia

**Keywords:** goat, implantation, melatonin, meta-analysis, reproduction, sheep

## Abstract

**Background and Aim:**

The impact of exogenous melatonin on the sperm quality of small ruminants is controversial. Therefore, this study aimed to synthesize previous findings on the influence of melatonin injection on sperm quality, steroid hormones, and testicular blood flow in small ruminants.

**Materials and Methods:**

Thirty studies were analyzed by computing the raw mean difference (RMD) as the effect size between the control and melatonin treatment groups, using the inverse of the variance for the random-effect model of the method of moments by DerSimonian and Laird. We assessed heterogeneity among studies using Q test. I^2^ statistic was used to classify the observed heterogeneity. We used Egger’s regression method to indicate publication bias.

**Results:**

Melatonin injection (p < 0.05) affected sperm concentration (RMD = 0.42 × 10^9^/mL), morphology (RMD = 2.82%), viability (RMD = 2.83%), acrosome integrity (RMD = 4.26%), and DNA integrity (RMD = 1.09%). Total motility (RMD = 5.62%), progressive motility (RMD = 7.90%), acrosome integrity (RMD = 8.68%), and DNA integrity (RMD = 2.01%) of post-thawed semen in the melatonin-treated group were also increased (p < 0.05). Similarly, treatment with melatonin (p < 0.05) enhanced total motility (RMD = 5.78%), progressive motility (RMD = 5.28%), curvilinear velocity (RMD = 4.09 μm/s), straight-line velocity (RMD = 5.61 μm/s), and average path velocity (RMD = 4.94 μm/s). Testosterone (RMD = 1.02 ng/mL) and estradiol 17-ß levels (RMD = 0.84 pg/mL) were elevated (p < 0.05) in the melatonin-injected group. Melatonin implantation ameliorated testicular blood flow, as indicated by a significant reduction (p < 0.05) in the resistive index (RMD = 0.11) and pulsatility index (RMD = –0.15).

**Conclusion:**

Melatonin administration can increase the reproductive performance of small male ruminants.

## Introduction

Reproductive inefficiency is a major threat to the sustainability of small ruminants, causing significant economic losses [1]. The reproductive efficacy of male animals, especially small ruminants, is equally important as that of female animals [2]. The quality of sperm accounts for reproductive success [3]. It contributes to 50% of the flock’s performance [4]. Effective management of small male ruminants before and during the breeding season is essential to reduce suboptimal reproductive performance and increase the profitability and sustainability of sheep production [5, 6]. However, only 70%–75% of rams exhibit peak reproductive performance at the beginning of the breeding season [7]. Therefore, it is necessary to devise a proper strategy to enhance the reproductive traits of small male ruminants.

Melatonin, a neurohormone secreted by the pineal gland, is a critical regulator of various physiological processes, including the regulation of circadian rhythms, antioxidant defenses, and immune modulation [8]. Melatonin administration has been intensively investigated to modulate reproductive cycles in small male ruminants. Melatonin injection has beneficial effects on testosterone production, sperm, and quality of post-thawed semen in sheep and goats during both breeding and non-breeding seasons [9–30]. Melatonin administration also mitigates sperm abnormalities in heat-stressed rams [31]. However, contradictory results have also been reported. Melatonin implantation in the ram does not correlate with sperm production or concentration [32]. Similarly, sperm quality and testosterone levels do not improve in melatonin-treated rams during the breeding season [33]. Furthermore, the administration of melatonin during breeding and non-breeding seasons has no impact on sperm and post-thawed semen quality, including motility and morphology in rams [34–37]. In addition, testosterone levels do not differ between the untreated and melatonin-treated rams during light challenges [38]. These contradictory findings necessitate a comprehensive statistical assessment to determine the influence of melatonin administration on sperm quality in small ruminants.

Meta-analysis is a statistical technique used to synthesize the results of previous studies to produce a robust quantitative conclusion [39]. It provides an unbiased and objective synthesis [40, 41]. To the best of our knowledge, no meta-analysis has been conducted on the relationship between melatonin implantation and sperm quality in small ruminants. Therefore, this meta-analysis synthesized the results of previous studies on the influence of melatonin injection on reproduction traits and sperm quality in small ruminants.

## Materials and Methods

### Ethical approval

**Figure-1:**
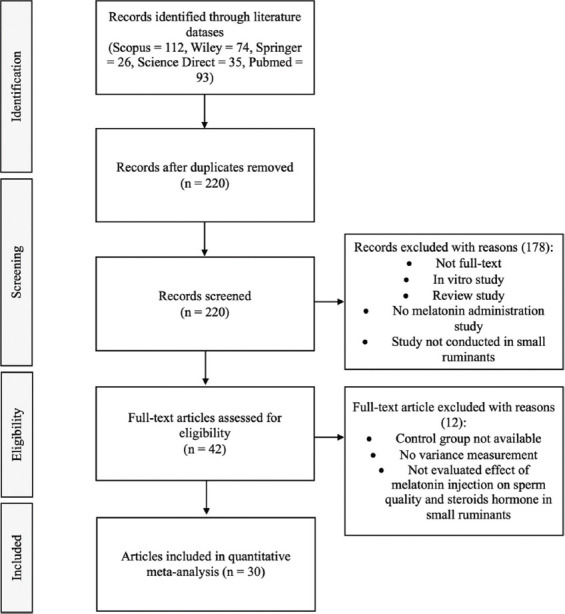
Selection of included studies using PRIMA protocols.

Ethical approval was not necessary for this study. The preferred reporting items for systematic review and meta-analyses (PRISMA) protocols were applied in this meta-analysis, as shown in Figure-1.

### Study period and location

The meta-analysis study was conducted from August to December 2023 at Faculty of Veterinary Medicine, Gadjah Mada University, Indonesia, and National Research and Innovation Agency, Indonesia.

### Search strategy

Comprehensive studies that assessed the impact of melatonin implantation on reproductive traits in small male ruminants were identified using the Science Direct, Wiley Online Library, PubMed, and Scopus databases. The search used the following keywords: “Melatonin”, “Sperm”, “Semen”, “Ram”, “Sheep”, “Buck”, and “Goat” which are connected through search queries such as “AND” and “OR”, respectively.

### Inclusion and exclusion criteria

After erasing the duplication, the identified studies were excluded if they were (1) *in vitro* studies, (2) review studies, (3) no full-text studies, (4) no small-ruminant studies, and (5) no melatonin implantation studies. Furthermore, the criteria for selected studies were as follows: (1) The control (non-treatment) group was available; (2) measures of variance (e.g., standard deviations [SD], standard errors [SE], or confidence intervals [CI]) were provided; and (3) the influence of melatonin injection on sperm quality, post-thawed semen quality, testicular blood flow, and plasma steroid hormones in small ruminants was studied.

### Extraction

The included studies are presented in Table-1 [9–38] as follows: First author’s name, year, location, species, season, dose, and research duration. The graphical data were extracted using WebPlotDigitizer (Automeris LLC, CA, USA) [42]. SD was calculated using the following formula: (1) SD = SE√N, where N is the repetition number; and (2) SD = √N × (upper CI–lower CI)/3.92, where 3.92 is the SE of the 95% CI and replaced with the t-distribution value if the sample was <60 in publications with no report of SD [43].

**Table-1:** The included studies.

No.	Author	Year	Location	Species	Season	Dose, mg	Duration, d
1	Abbas *et al*. [9]	2021	Pakistan	Goat	Non-breeding	18	70
2	Casao *et al*. [10]	2010	Spain	Sheep	Non-breeding	54	120
3	Casao *et al*. [11]	2013	Spain	Sheep	Non-breeding	54	147
4	Delgadillo *et al*. [12]	2001	Mexico	Goat	Non-breeding	36	315
5	Egerszegi *et al*. [13]	2013	Hungary	Sheep	Non-breeding	18,36	30
6	El-Shalofy *et al*. [14]	2021	Egypt	Sheep	Breeding	18	42
7	El-Shalofy *et al*. [15]	2022	Egypt	Sheep	Non-breeding	18,36	56
8	Gallego-Calvo *et al*. [16]	2015	Spain	Goat	Breeding and non-breeding	54	60
9	Hanif *et al*. [17]	1991	United Kingdom	Sheep	Non-breeding	90,118	60
10	Kleemann *et al*. [18]	2021	Australia	Sheep	Non-breeding	54	270
11	Kleemann *et al*. [19]	2022	Australia	Sheep	Non-breeding	18,36,54	120
12	Kokolis *et al*. [20]	2000	Greece	Sheep	Breeding and non-breeding	54	105
13	Leyva-Corona *et al*. [21]	2023	Mexico	Sheep	Non-breeding	18,36	120
14	Pool *et al*. [22]	2020	Australia	Sheep	Non-breeding	54	161
15	Rekik *et al*. [23]	2015	Jordan	Sheep	Non-breeding	54	60
16	Rosa *et al*. [24]	2000	United Kingdom	Sheep	Non-breeding	18,36	42
17	Roshan *et al*. [25]	2023	Iran	Sheep	Non-breeding	54	60
18	Samir *et al*. [26]	2020	Japan	Goat	Non-breeding	36	56
19	Tsantarliotou *et al*. [27]	2008	Greece	Sheep	Breeding and non-breeding	18	105
20	Vince *et al*. [28]	2017	Croatia	Goat	Non-breeding	72	90
21	Zarazaga *et al*. [29]	2010	Spain	Goat	Non-breeding	54	210
22	Shahat *et al*. [30]	2022	Canada	Sheep	Breeding	36	49
23	Shahat *et al*. [31]	2022	Canada	Sheep	Breeding	36	49
24	Rosa *et al*. [32]	2012	United Kingdom	Sheep	Non-breeding	18,36	42
25	Kaya *et al*. [33]	2000	Turkey	Sheep	Breeding and non-breeding	18,36	66
26	Buffoni *et al*. [34]	2015	Argentine	Sheep	Non-breeding	54	90
27	Faigl *et al*. [35]	2009	Hungary	Sheep	Non-breeding	54	71
28	Kaya *et al*. [36]	2001	Turkey	Sheep	Breeding and non-breeding	18	71
29	Pool *et al*. [37]	2020	Australia	Sheep	Breeding	54	210
30	Abecia *et al*. [38]	2017	Spain	Sheep	Breeding	18	30

### Statistical analysis

Meta-analysis and meta-regression were performed using the “metafor” package (Free Software Foundation, Inc., MA, USA) [44] and R software (R Foundation, Vienna, Austria) [45]. The influence of melatonin injection on reproductive traits and steroid hormones in small male ruminants was synthesized by computing the raw mean differences (RMDs) between untreated (control) and treated (melatonin injection) means, using the inverse of the variance for the random-effect model of the method of moments by DerSimonian and Laird [46]. RMD was selected to measure the findings in the original units [47].

### Heterogeneity evaluation

We assessed heterogeneity among studies using *Q* test [48]. The significance level was set at p ≤ 0.10. *I^2^* statistic was used to classify the observed heterogeneity, where *I^2^* values <25% imply low, 25%–50% denote moderate, and >50% represent high [49]. We used Egger’s regression method to indicate publication bias [50]. Significance was defined as p ≤ 0.05.

### subgroup analysis

Meta-regression was performed if the following criteria were fulfilled: (1) Heterogeneity was significant (p ≤ 0.10 or *I^2^* > 50%), (2) there was no publication bias (p-value of Egger’s test >0.05), and (3) the number of comparisons was >10 [47]. A categorical covariate was the type of season (breeding and non-breeding). In addition, days post-treatment (days) and doses of melatonin (mg) were employed as continuous covariates. We applied the method of moments proposed by DerSimonian and Laird [46] to perform meta-regression. Subgroup analysis was implemented to evaluate RMD in the presence of significant results (p ≤ 0.05) within categorical or continuous covariates. The doses of melatonin were 18, 36, 54, and more than 54 mg. Furthermore, the days post-injection were sub-grouped into 1–31, 31–60, and >60 days post-injection.

## Results

### Study attributes

Thirty studies were performed in 15 countries, primarily Spain (16.67%), Australia (13.33%), and the UK (10.00%) (Table-1) [9–38]. The percentages of sheep and goats were 80% and 20%, respectively. The studies were conducted during the breeding (28.57%) and non-breeding (71.43%) seasons. In addition, melatonin doses ranged from 18 to 118 mg and the duration ranged from 30 to 315 days.

### Quality of sperm and post-thawed semen

Melatonin treatment increased (p < 0.05) sperm concentration, normal morphology, viability, acrosome integrity, and DNA integrity (Table-2). All post-thawed sperm quality parameters, including progressive motility, total motility, acrosome, and DNA integrity, were increased (p < 0.05) in melatonin-injected small ruminants (Table-3).

**Table-2:** Effect of implanted melatonin on sperm quality of small-ruminants.

Item	N	Control means (SD)	RMD (95% CI)	p-value	Heterogeneity test		Egger’s test
	
					p-value	*I*^2^ (%)	p-value
Volume, mL	67	0.90 (0.30)	−0.03 (−0.08; 0.03)	ns	<0.0001	78.14	0.099
Concentration, ×10^9^/mL	65	3.68 (1.30)	0.42 (0.24; 0.58)	<0.0001	<0.0001	74.94	0.047
Cell number, ×10^9^/mL	29	3.62 (1.57)	0.33 (−0.18; 0.84)	ns	<0.0001	81.97	0.413
Mass motility, score 1–5	41	3.19 (1.05)	−0.08 (−0.23; 0.06)	ns	<0.0001	84.64	0.002
Live sperm, %	44	71.63 (9.43)	0.65 (−0.40; 1.71)	ns	<0.0001	73.53	<0.0001
Normal morphology, %	50	75.03 (5.35)	2.82 (1.22; 4.42)	<0.001	<0.0001	88.44	0.850
Viability, %	33	71.60 (5.07)	2.83 (1.68; 4.00)	<0.0001	<0.001	50.22	0.070
Acrosome integrity, %	21	82.47 (1.79)	4.26 (2.14; 6.37)	<0.0001	<0.0001	96.65	0.100
DNA integrity, %	25	92.87 (4.26)	1.09 (0.70; 1.49)	<0.0001	0.474	0.00	0.032

N=Number of comparisons, SD=Standard deviation, CI=Confidence of interval, *I*^2^=Inconsistency index, ns=Non-significant, RMD=Raw mean difference

**Table-3:** Post-thawed semen quality between melatonin-treated and control small-ruminants.

Item	N	Control means (SD)	RMD (95% CI)	p-value	Heterogeneity test		Egger’s test
	
					p-value	*I*^2^ (%)	p-value
Post-thawed total motility, %	16	50.91 (11.49)	5.62 (2.28; 8.95)	<0.001	<0.0001	76.65	0.041
Post-thawed progressive motility, %	15	32.34 (10.56)	7.90 (3.08; 12.72)	0.001	<0.0001	90.73	0.810
Post-thawed acrosome integrity, %	9	66.13 (2.94)	8.68 (4.23; 13.12)	<0.001	<0.0001	95.98	0.782
Post-thawed DNA integrity, %	11	68.35 (1.08)	2.01 (0.65; 3.37)	0.004	<0.0001	97.04	0.207

N=Number of comparisons, SD=Standard deviation, CI=Confidence of interval, *I*^2^ =Inconsistency index,

ns=Non-significant, RMD=Raw mean difference

### Sperm kinetic parameters

Total motility in melatonin-treated small ruminants increased (p < 0.05). Similarly, melatonin treatment enhanced average path velocity (VAP), straight-line velocity (VSL), curvilinear velocity (VCL), and progressive motility (Table-4). However, melatonin did not influence the amplitude of lateral head displacement (p < 0.05).

**Table-4:** Comparison results of sperm kinetic parameters in melatonin-treated and control small-ruminants.

Item	N	Control means (SD)	RMD (95% CI)	p-value	Heterogeneity test		Egger’s test
	
					p-value	*I*^2^ (%)	p-value
Total motility, %	27	74.40 (5.40)	5.78 (2.32; 9.23)	0.001	<0.0001	98.41	0.789
Progressive motility, %	57	52.86 (4.75)	5.28 (3.85; 6.70)	<0.0001	<0.0001	99.72	0.007
VCL, µm/s	33	87.89 (9.07)	4.09 (2.39; 5.79)	<0.0001	<0.0001	85.45	0.080
VSL, µm/s	33	63.27 (7.34)	5.61 (2.42; 8.91)	0.001	<0.0001	88.69	0.823
VAP, µm/s	33	61.80 (7.66)	4.94 (2.08; 7.81)	0.001	<0.0001	87.67	0.461
ALH, µm	23	2.42 (0.22)	0.02 (−0.04; 0.07)	ns	0.039	37.16	0.962

N=Number of comparisons, SD=Standard deviation, CI=Confidence of interval, *I*^2^=Inconsistency index, ns=Non-significant, VCL=Curvilinear velocity, VSL=Straight-line velocity, VAP=Average path velocity, ALH=Amplitude of lateral head displacement, RMD=Raw mean difference

### Reproductive steroid hormones

Testosterone levels were increased (p < 0.05) in small melatonin-implanted ruminants (Table-5). In addition, the administration of melatonin improved (p < 0.05) estradiol 17-ß levels.

**Table-5:** Effect of exogenous melatonin on reproductive steroid hormones in male small-ruminants.

Item	N	Control means (SD)	RMD (95% CI)	p-value	Heterogeneity test		Egger’s test
	
					p-value	*I*^2^ (%)	p-value
Testosterone, ng/mL	209	5.68 (2.62)	1.02 (0.70; 1.35)	<0.0001	<0.0001	92.88	<0.0001
Estradiol 17-ß, pg/mL	40	66.18 (13.55)	0.84 (0.13; 1.54)	0.020	<0.0001	54.87	<0.0001

N=Number of comparisons, SD=Standard deviation, CI=Confidence of interval, *I*^2^=Inconsistency index, ns=Non-significant, RMD=Raw mean difference

## Testicular blood flow

Melatonin injection affected the resistive index (RI), peak systolic velocity (PSV), and pulsatility index (PI) (p < 0.05) (Table-6). End-diastolic velocity was not influenced by melatonin administration (p > 0.05).

**Table-6:** Effect of injected melatonin on testicular blood flow parameters (pulsed-wave Doppler indices) of small-ruminants.

Item	N	Control means (SD)	RMD (95% CI)	p-value	Heterogeneity test		Egger’s test
	
					p-value	*I*^2^ (%)	p-value
PSV, cm/s	34	26.23 (5.94)	−2.63 (−4.24; −1.02)	0.001	<0.0001	71.08	0.232
EDV, cm/s	34	12.09 (4.06)	−0.39 (−1.18; 0.39)	ns	0.043	31.53	0.119
RI	42	0.55 (1.85)	−0.11 (−0.13; −0.09)	<0.0001	0.017	34.36	0.499
PI	42	0.79 (2.19)	−0.15 (−0.18; −0.10)	<0.0001	0.002	42.62	0.750

N=Number of comparisons, SD=Standard deviation, CI=Confidence of interval, *I*^2^=Inconsistency index, ns=Non-significant, PSL=Peak systolic velocity, EDV=End-diastolic velocity, RI=Resistive index, PI=Pulsatility index, RMD=Raw mean difference

### Publication bias and meta-regression analysis

Heterogeneity was significant (p < 0.05) for all parameters of sperm quality, post-thawed semen quality, sperm motility, reproductive steroid hormones, and testicular blood flow parameters (Tables-2-6). On the other hand, publication bias was not found (p > 0.05) on sperm normal morphology, sperm total motility, sperm viability, acrosome integrity, VCL, VSL, VAP, PSV, RI, PI, post-thawed progressive motility, post-thawed acrosome integrity, and post-thawed DNA integrity. Meta-regression was performed when the effect size and heterogeneity were significant and publication bias was not present.

The melatonin dose explained 65.17%, 100%, 93.79%, and 47.71% of the observed heterogeneity for acrosome integrity, RI, PI, and VCL, respectively (p < 0.0001) (Table-7). Days post-melatonin treatment explained 58.00% (p < 0.001), 26.96% (p = 0.034), and 44.64% (p = 0.006) of the observed heterogeneity for sperm viability, RI, and PI, respectively. Moreover, melatonin dose explained 31.71% (p = 0.037), 36.53% (p = 0.001), an d 5.44% (p = 0.002) of the observed heterogeneity for VAP, post-thawed progressive motility, and post-thawed DNA integrity.

**Table-7:** Meta-regression comparing the association between covariates and measured outcomes.

Parameter	Covariates	QM	p-value	R^2^ (%)
Sperm normal morphology	Dose	0.01	ns	1.32
	Days post-injection	2.57	ns	6.39
	Season	21.03	<0.0001	24.97
Sperm viability	Dose	1.10	ns	8.44
	Days post-injection	14.20	<0.001	58.00
	Season	*NA*	*NA*	*NA*
Sperm total motility	Dose	0.16	ns	0
	Days post-injection	0.27	ns	16.73
	Season	4.26	0.040	45.54
Acrosome integrity	Dose	34.59	<0.0001	65.17
	Days post-injection	0.20	ns	0
	Season	6.58	0.010	0
PSV	Dose	0	ns	0
	Days post-injection	0.10	ns	0
	Season	0.39	ns	0
RI	Dose	27.53	<0.0001	100
	Days post-injection	4.47	0.034	26.96
	Season	2.19	ns	12.14
PI	Dose	28.09	<0.0001	93.79
	Days post-injection	7.59	0.006	44.64
	Season	35.80	<0.0001	100
VCL	Dose	31.62	<0.0001	47.71
	Days post-injection	3.15	ns	0
	Season	41.28	<0.0001	49.37
VSL	Dose	1.46	ns	17.02
	Days post-injection	0.002	ns	0
	Season	16.79	<0.0001	32.35
VAP	Dose	4.34	0.037	31.74
	Days post-injection	0.24	ns	0
	Season	18.25	<0.0001	45.94
Post-thawed progressive motility	Dose	11.42	<0.001	36.53
	Days post-injection	0.05	ns	0
	Season	10.60	0.001	35.90
Post-thawed DNA integrity	Dose	4.76	0.002	5.44
	Days post-injection	1.86	ns	36.91
	Season	4.76	0.002	5.44

QM=Coefficient of moderators, R^2^=Amount of heterogeneity accounted for by covariate, NA=Non-available, ns=Non-significant, PSV=Peak systolic velocity, RI=Resistive index, PI=Pulsatility index, VCL=Curvilinear velocity, VSL=Straight-line velocity, VAP=Average path velocity

The season of the experiment explained (p < 0.001) 24.97%, 100%, 49.37%, 32.35%, and 45.94% for normal sperm morphology, PI, VCL, VSL, and VAP, respectively. In addition, 35.39% (p = 0.001) and 5.44% (p = 0.002) of the observed heterogeneity for post-thawed progressive motility and post-thawed DNA integrity were explained by the season of the experiment (Table-7).

### subgroup analysis

**Figure-2:**
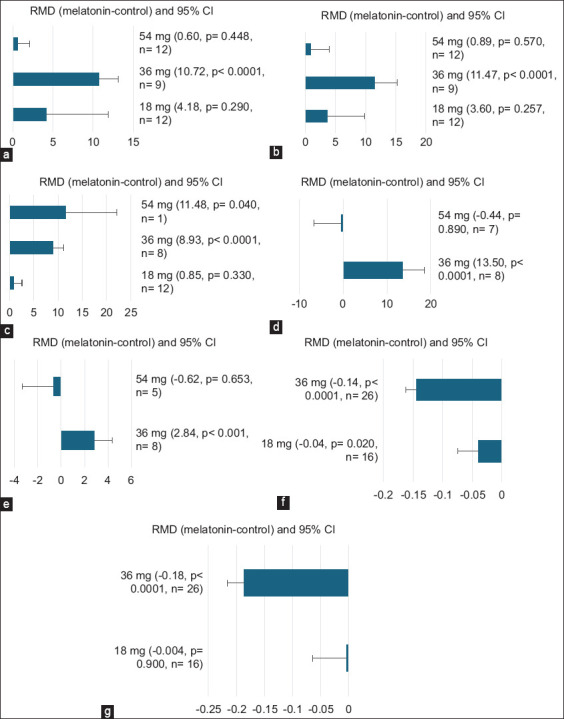
Subgroup analysis of the effect of dose melatonin injection on (a) straight-line velocity, μm/s, (b) average path velocity, μm/s, (c) acrosome integrity, %, (d) post-thawed progressive motility, %, (e) post-thawed DNA integrity, %, (f) resistive index, and (g) pulsatility index.

The dose of 36 mg melatonin increased (p < 0.0001) VCL (Figure-2a; RMD = 10.72), VAP (Figure-2b; RMD = 11.74), acrosome integrity (Figure-2c; RMD = 8.93), post-thawed progressive motility (Figure-2d; RMD = 13.50), and post-thawed DNA integrity (Figure-2e; RMD = 2.84). Similarly, 54 mg dose enhanced acrosome integrity (p = 0.04) (Figure-2c; RMD = 11.48). However, 18- and 36-mg melatonin reduced RI (RMD = –0.04; p = 0.02 and RMD = –0.14; p < 0.0001, respectively) (Figure-2f). PI decreased (p < 0.0001) at 36 mg injection of melatonin (Figure-2g; RMD = –0.18).

**Figure-3:**
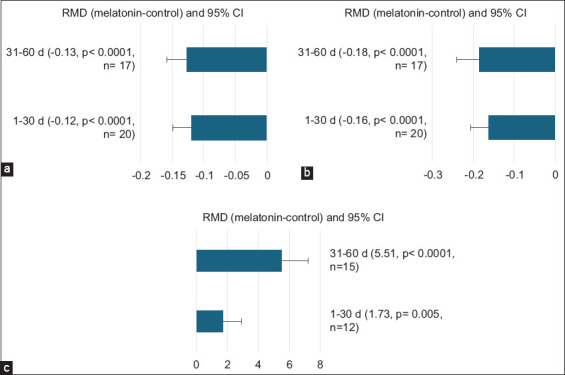
Subgroup analysis of the season type of effect of melatonin administration on (a) resistive index, (b) pulsatility index, and (c) sperm viability, %.

RI was reduced (p < 0.0001) on days 1–30 post-melatonin treatment (RMD = –0.12) and 31–60 post-melatonin treatment (RMD = –0.13) (Figure-3a). Similarly, PI decreased (p < 0.0001) on 1–30 days (RMD = –0.16) and 31–60 days post-melatonin treatment (RMD = –0.18) (Figure-3b). Sperm viability was enhanced on days 1–30 (RMD = 1.73; p = 0.005) and 31–60 (RMD = 5.51; p < 0.0001) post-melatonin treatment (Figure 3c).

Melatonin injection during the breeding season enhanced (p < 0.0001) normal sperm morphology (RMD = 9.70), sperm total motility (RMD = 8.57), acrosome integrity (RMD = 7.83), VCL (RMD = 9.73), VSL (RMD = 13.90), VAP (RMD = 11.34), post-thawed progressive motility (RMD = 12.46), and post-thawed DNA integrity (RMD = 2.84) (Table-8). Moreover, melatonin implantation decreased PI (p < 0.0001) in the breeding (RMD = –0.09) and non-breeding (RMD = –0.27) seasons.

**Table-8:** Subgroup analysis of season type of the effect of melatonin injection on sperm quality, sperm motility, and testicular blood flow parameters.

Parameter	Breeding			Non-breeding		
	
	N	RMD (95% CI)	p-value	N	RMD (95% CI)	p-value
Sperm normal morphology	9	9.70 (6.44; 12.96)	<0.0001	41	1.22 (−00.36; 2.80)	ns
Sperm total motility	11	8.57 (4.80; 12.34)	<0.0001	16	3.02 (−00.65; 6.70)	ns
PI	15	−0.09 (−0.13; −0.07)	<0.0001	27	−00.27 (−032; −00.22)	<0.0001
Acrosome integrity	9	7.83 (4.22; 11.43)	<0.0001	12	1.54 (−01.63; 4.71)	ns
VCL	9	9.73 (7.44; 12.02)	<0.0001	24	0.48 (−01.18; 2.13)	ns
VSL	9	13.90 (9.09; 18.71)	<0.0001	24	1.62 (−01.74; 4.99)	ns
VAP	9	11.34 (7.62; 15.07)	<0.0001	24	1.08 (−01.79; 3.96)	ns
Post-thawed progressive motility	9	12.46 (7.63; 17.30)	<0.0001	6	−01.67 (−08.67; 5.33)	ns
Post-thawed DNA integrity	8	2.84 (1.32; 4.37)	<0.001	3	−00.62 (−03.34; 2.09)	ns

N=Number of comparisons, RMD=Raw mean difference, CI=Confidence of interval, ns=Non-significant, PI=Pulsatility index, VCL=Curvilinear velocity, VSL=Straight-line velocity, VAP=Average path velocity

## Discussion

Melatonin modulates the release of gonadotropin-releasing hormone [51], a key regulator of reproductive physiology. It is actively transported into the testes [52], modulating various cellular processes involved in spermatogenesis and steroidogenesis [53, 54]. Melatonin administration enhances spermatogenesis [55].

Our meta-analysis revealed that treatment with melatonin enhances sperm quality in small ruminants, including viability, concentration, normal morphology, acrosome, and DNA integrity. These findings are similar to those reported by Abbas *et al*. [9], who reported that injection of melatonin increases the concentration, normal morphology, acrosome, and DNA integrity of sperm in rams. Moreover, motility, acrosome, and DNA integrity in the post-thawed semen of melatonin-treated small ruminants were also increased. Shahat *et al*. [30] discovered that injection of melatonin improves post-thawed motility, acrosome, and DNA integrity of rams.

Blood circulation is crucial, especially for testicular function [56]. Enhanced testicular blood flow increases the supply of oxygen and nutrients to the testes [57]. Melatonin enhances testicular blood flow [26] and is correlated with heightened sperm quality in rams [31]. Resistance (RI) and perfusion (PI) indices are key indicators extensively used for evaluating testicular blood flow in various animals [58–61]. Reduced RI and PI values indicated elevated testicular blood flow [62]. This study also found that elevated sperm quality is linked to low RI and PI values. Therefore, the increase in sperm and post-thawed semen quality in melatonin-treated small ruminants can be attributed to the enhancement of testicular blood supply.

This study showed that melatonin implantation improves sperm motility and velocity of small ruminants. Egerszegi *et al*. [13] and Casao *et al*. [10] also found that injection of melatonin increases sperm motility and ram velocity in the out-of-season period. Moreover, Shahat *et al*. [31] found that sperm motility and velocity of melatonin-treated rams subjected to mild heat-stressed challenges increased during breeding seasons.

RI and PI are negatively correlated with the progressive motility of sperm [62]. Decreased arterial blood flow to the testes impairs mitochondrial energy processes, preventing spermatogenesis. This dysfunction of the energetic pathway reduces sperm motility [63]. Our results revealed that sperm kinetic parameters increased concomitantly with the enhancement of testicular blood flow. Thus, we suggest that melatonin modulates testicular blood flow, resulting in increased mitochondrial energy production and enhanced sperm motility.

In this study, we found that testosterone and estradiol-17β levels are elevated in melatonin-treated small ruminants. These findings are consistent with those of previous studies where testosterone levels were elevated in melatonin-treated rams and bucks [16, 31]. Melatonin affects testosterone production through the anterior pituitary gland and ameliorates Leydig cell function to promote testosterone secretion in sheep [64]. Samir *et al*. [26] found that melatonin elevates testosterone production in Shiba bucks through the hypothalamus-pituitary axis. Moreover, Casao *et al*. [10] discovered that the enhancement of antioxidant enzymes induces the elevation of testosterone and estradiol-17β levels in melatonin-treated rams.

Furthermore, our study demonstrated that melatonin implantation increases PSV and decreases RI and PI, indicating enhanced testicular blood supply. RI and PI levels are regulated by plasma estradiol-17β [65]. Bollwein *et al*. [66] found that estradiol-17β levels, but not testosterone levels, control testicular blood flow in stallions. Moreover, Salama *et al*. [67] discovered that enhanced testicular blood flow in melatonin-treated canines is triggered by elevation of estradiol-17β levels. Similarly, El-Shafoly *et al*. [15] reported that melatonin-injected rams show a reduction in RI and PI levels accompanied by an increase in estradiol-17β levels. Interestingly, our study also demonstrated a correlation between decreased RI and PI and enhancement of estradiol-17β levels. Therefore, we suggest that melatonin administration improves testicular blood flow in small ruminants by modulating estradiol-17β levels.

## Conclusion

These results indicate that melatonin administration can improve sperm quality in small ruminants. The best outcomes for acrosome integrity, VCL, post-thawed progressive motility, and post-thawed DNA integrity were achieved with 36 mg melatonin injection during the breeding season. As evidenced by a reduction in RI and PI, optimal results for testicular blood supply were attained with 36 mg melatonin implantation during the breeding season within 1–60 days post-injection. Moreover, melatonin administration was associated with superior results in normal morphology, motility, VSL, and VAP during the breeding season.

## Authors’ Contributions

AB: Designed the study and wrote and revised the manuscript. SH: Screened studies, analyzed data, and edited the manuscript. RW: Scrutinized included studies and edited and reviewed manuscript. HK, WW, BH, IMM, AI, and DDL: Extracted data and reviewed the manuscript. All authors have read, reviewed, and approved the final manuscript.
